# Determination of caspase-3 activation fails to predict chemosensitivity in primary acute myeloid leukemia blasts

**DOI:** 10.1186/1471-2407-5-60

**Published:** 2005-06-11

**Authors:** Peter Staib, Jan Tiehen, Timo Strunk, Timo Schinköthe

**Affiliations:** 1 Clinic I for Internal Medicine, The University Hospital of Cologne, Kerpener Str. 62, 50924 Cologne, Germany

## Abstract

**Background:**

Ex-vivo chemosensitivity tests that measure cell death induction may predict treatment outcome and, therefore, represent a powerful instrument for clinical decision making in cancer therapy. Such tests are, however, work intensive and, in the case of the DiSC-assay, require at least four days. Induction of apoptosis is the mode of action of anticancer drugs and should, therefore, result in the induction of caspase activation in cells targeted by anticancer therapy.

**Methods:**

To determine, whether caspase activation can predict the chemosensitivity, we investigated enzyme activation of caspase-3, a key executioner caspase and correlated these data with chemosensitivity profiles of acute myeloid leukemia (AML) blasts.

**Results:**

There was, however, no correlation between the ex-vivo chemosensitivity assessed by measuring the overall rates of cell death by use of the DiSC-assay and caspase-3 activation.

**Conclusion:**

Thus, despite a significant reduction of duration of the assay from four to one day, induction of apoptosis evaluated by capase-3 activity does not seem to be a valid surrogate marker for chemosensitivity.

## Background

Cytogenetic abnormalities, age, initial leucocyte count and P-glycoprotein expression are examples of widely accepted prognostic factors in acute myeloid leukemia [1-5]. However, the prognosis of an individual patient may still not accurately be determined by these factors. The ex-vivo chemosensitivity profile may help to predict the individual response to therapy and, more importantly, to individualize the treatment and to identify new agents [6-8].

Many ex-vivo chemosensitivity assays have been investigated for over 40 years. The clonogenic assay is a long term assay but technical drawbacks and the long culturing time have limited the clinical use of the method [9,10]. Most assays used today are short term total cell kill assays, however, the methods to determine viable cells after incubation with cytotoxic agents vary greatly between direct evaluation of the cells and, in the vast majority, indirect evaluation using different surrogate markers. The differential staining cytotoxicity assay (DiSC) uses microscopic evaluation of dye exclusion in viable cells [11,12]. In the MTT assay, surviving cells reduce the methyl-thiazol-tetrazolium (MTT) into highly colored formazan which can be quantified by spectrophotometry [13-15]. Other assays have used fluorescein diacetate (FMCA) or cellular ATP content as a surrogate marker of cell viability [16,17]. All these assays mentioned above are based on essentially the same culturing procedures in terms of incubating fresh leukemic cells with cytotoxic agents over a period of four to five days. A shorter duration of assays, however, may be useful to obtain ex-vivo chemosensitivity profiles prior the onset of treatment and would allow to individualize the therapy in most patients.

It is generally accepted that cytotoxic drugs eliminate malignant cells by inducing apoptosis (programmed cell death) [18,19]. This process starts, as it was shown in hematopoietic tumor cell lines, within hours of exposure to cytotoxic drugs [20]. Thus, quantification of a surrogate marker of apoptosis might be a suitable method for a fast ex-vivo chemosensitivity assay. The appearance of phospholipids like phosphatidylserine at the cell surface is a common marker for apoptosis which can be detected by Annexin V using flow cytometry [21,22]. Apoptosis may also be quantified by changes of the mitochondrial electrochemical transmembrane potential using DiOC_6 _or JC-1 [21,23], by detection of DNA- respectively nuclear-fragmentation using the TdT-assay [23], gel-electrophoresis or acridine orange [21,24-26]. Further apoptotic markers are the cleaved forms of poly (ADP-ribose) polymerase (PARP) and caspase-3 which both are detectable by specific antibodies [24,25,27-29].

Most of these methods allow some grade of automation and a reasonable number of samples to be processed, which is needed for the clinical use of an ex-vivo chemosensitivity assay as well as for the identification of new agents. A development allowing more automation and larger sample sizes might be the detection of activated caspase-3, the main effector caspase in the apoptotic enzyme cascade, by using fluorogenic substrates and detection of enzyme activation in microplate readers [30]. To evaluate the usefulness and accuracy of such a procedure, we investigated the ex-vivo chemosensitivity assessed by measuring caspase-3 activation and, also, compared these data to results obtained by DiSC assay in AML blasts.

## Methods

### Patient samples

Bone marrow (BM) or peripheral blood (PB) were taken from adult patients with acute myeloid leukemia (AML) with informed consent during routine diagnostic BM aspiration and/or phlebotomy before treatment was started. Samples were collected in heparinized tubes from patients treated at our institution.

### Cell lines

For establishing the caspase-3 assay the AML cell lines THP-1, HEL and KG-1 were used.

### Drugs

Drugs were dissolved according to manufacturers' instructions and diluted in RPMI 1640 culture medium (Gibco, Paisley, UK). In order to induce cytotoxicity levels ex vivo comparable with those induced in vivo, the drugs were used at concentrations encompassing the range of clinically relevant plasma levels, as previously reported [31]. Cytarabine (Ara-cell™, cell-pharm, Hannover, Germany) was used at final concentrations in the range from 0.06 μg/ml to 5 μg/ml in the DiSC-Assay and from 0.06 to 20 μg/ml in the caspase-3 assay. Daunorubicin (Daunoblastin™, Pharmacia, Erlangen, Germany) was tested at final concentrations ranging from 0.008 μg/ml to 2 μg/ml and from 0.008 μg/ml to 5 μg/ml, mitoxantrone (Novantron™, Lederle, Münster, Germany) from 0.006 μg/ml to 0.5 μg/ml and from 0.006 μg/ml to 20 μg/ml in the DiSC-assay and the caspase-3 assay, respectively. The drugs were tested in duplicate samples at 5 different concentrations in the DiSC-Assay and in triplicate samples at six different concentrations in the caspase-3 assay.

### Differential Staining Cytotoxicity (DiSC)-Assay

The DiSC-assay was performed as previously described with minor modifications [11,32-34]. After density gradient isolation 10^5 ^fresh leukemic cells were resuspended in RPMI 1640 medium (Life Technologies, Germany) containing 10% heat-inactivated fetal calf serum (Biochrom, Germany) and penicillin-streptomycin (Life Technologies, Germany). Cells were incubated either without (control samples) or with added cytotoxic drug in a total volume of 200 μl (37°C, humidified 5% CO_2_) for 96 hours. After the incubation 1% fast-green and 0.5% nigrosin (Sigma Aldrich Chemie, Taufkirchen, Germany), including fixed duck erythrocytes as an internal standard, were added to each tube. Cells were transferred to collagen surfaced microscope slides by cytocentrifugation, air-dried and counterstained with May-Grünwald-Giemsa stain (Sigma Aldrich Chemie). Subsequent evaluation of slides by light microscopy facilitated the determination of drug efficacy at each drug concentration compared with controls.

### Caspase-3 activity assay

For measuring the activation of the caspase-3 we used the fluorometric assay TIA (Test for Induced Apoptosis) Product No. CDC_101 Evotec technologies, subsequently called Casp3-test (Evotec Analytical Systems, Erkrath, Germany). This assay employs the peptide substrate DEVD-AMC. 10^5 ^ficoll isolated mononuclear cells were incubated without (control samples) or with added cytotoxic drug in a total volume of 100 μl (37°C, humidified 5% CO_2_) for 16 hours. Afterwards, cell lysis was performed by adding 10 μl of lysis-solution (Evotec) and 90 μl lysis buffer (Evotec) to each well and incubating the suspension for one hour at room temperature in the dark on a shaker. For fluorometric detection of caspase-3 activity 1% (v/v) substrate DEVD covalently linked to the fluorogenic dye (7-amino-4-methyl coumarin, AMC) was added. Upon cleavage by caspase-3 free dye as an indicator for cumulated caspase-3 activity was detected using a fluorescence microplate reader (SPECTRAFluor, Tecan, Grödig/Salzburg, Austria) with a 360 nm excitation and a 465 nm emission filter for 30 to 60 minutes.

### Data analysis

For the DiSC assay logistic curves were fitted to the cell count survival data; the logit of survival probability was taken to be linear with respect to the logarithm of drug concentration [8,34,35]. LC_90_-doses, that is, the concentration of drug to produce a 90% reduction in cell survival compared with control cells, were determined by calculating the log dose at which the fitted survival probability was equal to 0.1. As LC_90_-results are log normal, all LC_90_-values were logged (base 10) before calculation of median, ranges of concentrations and statistical tests.

Established analyzing procedures for ex-vivo chemosensitivity assays assume that the minimum and the maximum drug-effects are defined. In case of enzymes like the caspases the in-vivo maximum activity in live cells cannot be reliably obtained. Therefore, an analyzing procedure to describe the relationship between drug concentration and caspase activation had to be developed for the Casp3-test and is presented as part of the results. For comparison between DiSC assay and Casp3-test results the Spearman's regression was applied.

## Results

### Patient characteristics

In this study, we examined 42 AML-specimens. Patient characteristics are shown in Table 1. The diagnosis and classification of AML was confirmed by central review of bone marrow slides, at our institution patients were treated according to treatment protocols of the AML-Cooperative Group (AMLCG, Germany).

### Evaluation of caspase-3 assay (Casp3-test)

**Figure 1:**
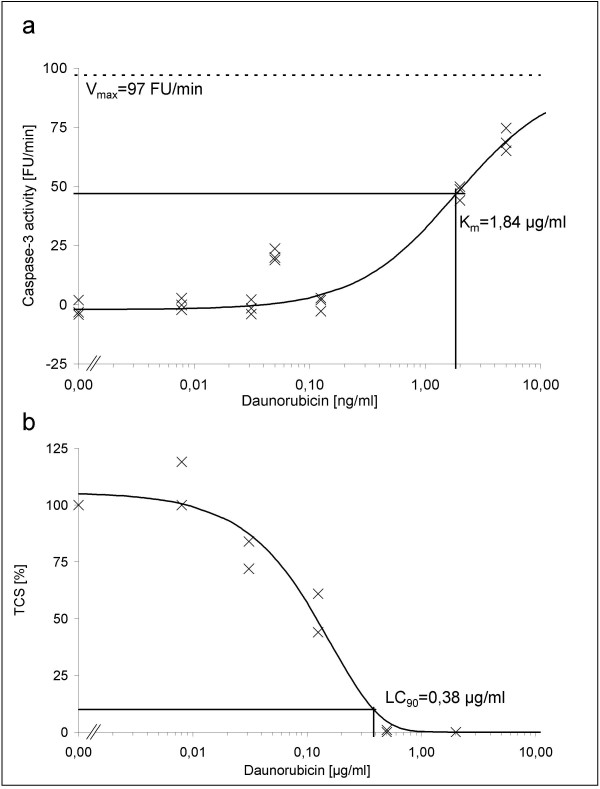
Example of a typical dose-response curve received by the Casp3-test (a) and the DiSC-assay (b) in leukemic cells from a patient with AML after incubation with daunorubicin. FU = Fluorescence Units, TCS = tumor cell survival.

Before using the Casp3-test for the assessment of the ex-vivo drug sensitivity an analyzing procedure had to be developed. With increasing drug concentrations we observed an increasing fluorescence signal following an S-shaped curve for the caspase-3 activity. This dependence of fluorescence upon the concentration of a cytotoxic drug with the appearance of a saturation behavior corresponds to kinetics described by the Michaelis-Menten equation. The base-level of fluorescence was integrated into the Michaelis-Menten equation by adding a constant "CutOff " as follows:

V = CutOff + V_max_*[S]/([S]+K_m_)

with V being the gradient of fluorescence, V_max _being the maximum gradient of fluorescence, [S] the drug concentration, CutOff the base-level of fluorescence and K_m _being the Michaelis constant describing the drug concentration at half maximum rate of reaction (Figure 1). The K_m _served as a measure for the cytotoxic efficacy of the chemotherapeutic agents.

For defining an optimal incubation period to allow time for all drugs studied to exert their apoptotic effect and, also, to find stable caspase-3 activity rates we performed time course experiments. The AML cell lines HEL, THP-1 and KG-1 were incubated with daunorubicin (0.03 – 5 μg/ml) and cytarabine (0.19 – 20 μg/ml). The incubation was stopped after every two hours up to 24 hours. Plotting the fluorescence signal in dependence upon incubation time, maximum values were observed at different time points corresponding to different drug concentrations. Usually, the highest drug concentrations achieved their maximum of relative fluorescence (compared to controls) after 8 to 12 hours of incubation in contrast to lower concentrations with 12 to 14 hours. Generally, higher fluorescence levels were obtained by higher drug concentrations.

### Stability of K_m_

**Figure 2:**
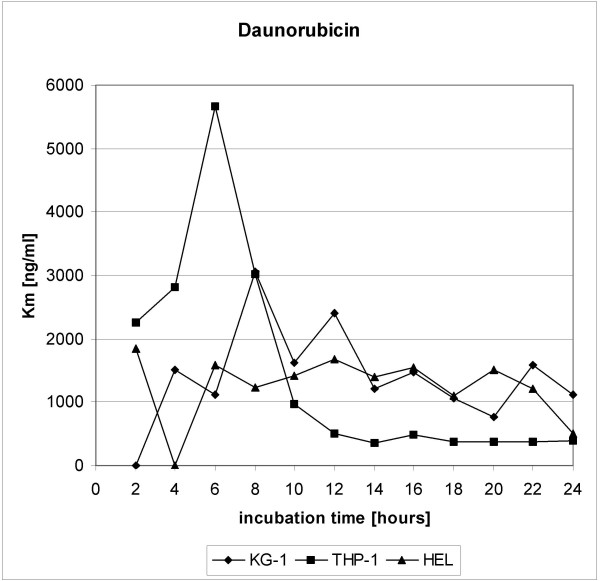
Average K_m_-values of daunorubicin and incubation time in the cell lines HEL, THP-1 and KG-1.

The K_m _as a marker for the cytotoxic efficacy of a specific drug was determined according to the Michaelis-equation as described above. Stable K_m_-values were achieved during incubation with daunorubicin within 14 hours and with cytarabine within 12 hours (Figure 2). In order to have reliably stable rates of apoptosis we chose an incubation time of 16 hours.

### Ex-vivo chemosensitivity by the DiSC-assay

In 37 of 42 AML samples (88%) the DiSC-assay was successfully performed. In 5 cases the assay failed due to bacterial contamination. All chemotherapeutic drugs tested – ara-C, daunorubicin and mitoxantrone – induced cell death in a dose-dependent manner (Figure 1). The sensitivities of the AML cells expressed as LC_90_s showed variability between the patients from as much as 22-fold below (for ara-C) to 105-fold above (also for ara-C) the median value (Table 2). For each drug, a range of sensitivities was observed, ranging from very sensitive to very resistant [11,36]. These individual patient drug sensitivity profiles reflect the patient heterogeneity seen in the clinic [37]. If more than 90% of the tumor cells were already killed by the lowest drug concentration, the LC_90_-value was set equal to the corresponding lowest drug concentration [13]. This was observed in seven cases after incubation with cytarabine.

### Ex-vivo chemosensitivity by the Casp3-test

The Casp3-test was successful in all 42 AML samples in terms of measurable caspase activity. But as far as the various cytotoxic drugs – ara-C, daunorubicin and mitoxantrone – are concerned apoptosis could be demonstrated in a dose-dependent manner only in a subset of 116 of 126 tests (92%) (Figure 1). The sensitivities of the AML blasts expressed as K_m _showed extreme variability between the patients from as much as 40-fold below (for daunorubicin) to 6,3 × 10^8^-fold above (for ara-C) the median value (Table 2). In 14 cases K_m_-values were less than the lowest concentration of ara-C used in the assay, hence the K_m_-values was set equal to the lowest ara-C concentration tested in these cases.

### Comparison between DiSC-assay and Casp3-test

Both assays were simultaneously performed on each of the 42 AML samples. Correlation between the two tests was possible for cytarabine in 33 cases, for daunorubicin in 36 cases and for mitoxantrone in 33 cases.

**Figure 3:**
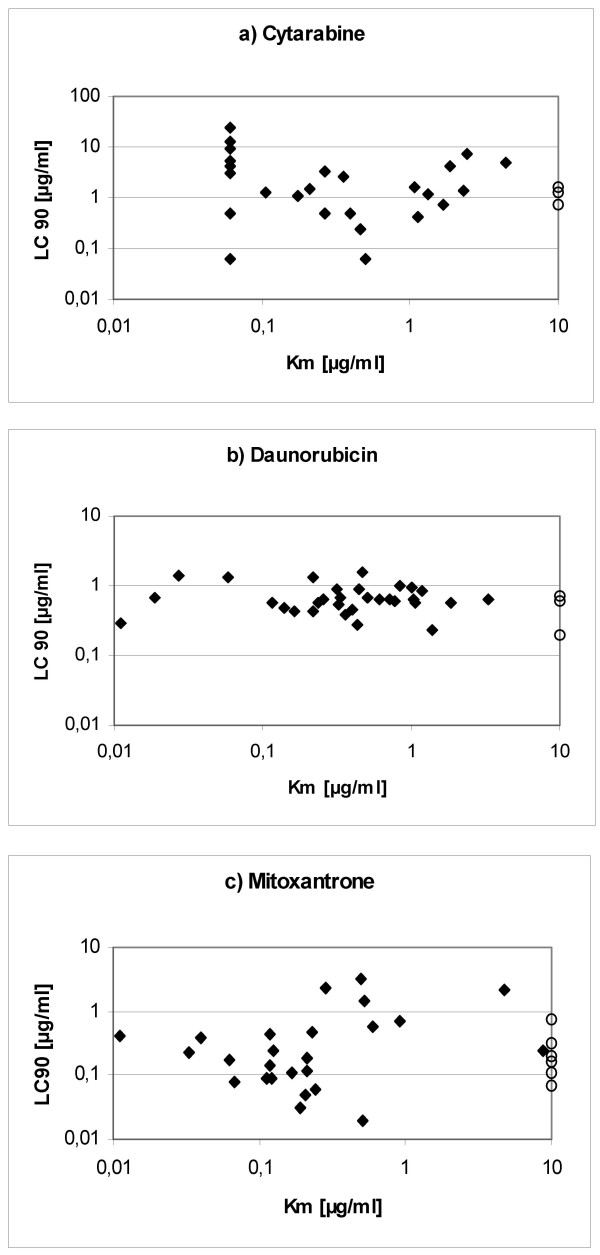
Distribution of the LC_90_- (DiSC-assay) and K_m_-values (Casp3-test) for the tested drugs a) cytarabine (N = 33), b) daunorubicin (N = 36), and c) mitoxantrone (N = 33).

For each drug tested the K_m_-values by the Casp3-test showed an extreme variability as compared to the LC_90_-values by the DiSC-assay, although the median values were quite similar (Table 2). LC_90_-values did not correlate with corresponding K_m_-values of cytarabine (R^2 ^= 0.38, p = 0.491), daunorubicin (R^2 ^= 0.001, p = 0.964) or mitoxantrone (R^2 ^= 0.005, p = 0.544) according to the regression after Spearman-Rho. The associations of LC_90_-values with K_m_-values of the three drugs are shown in Figure 3.

## Discussion

The major goal of this study was to determine whether the ex-vivo drug resistance in fresh leukemic cells from patients with AML is reliably assessed by a short-time apoptosis-assay that measures caspase-3 activation. To this end, we compared the Casp3-test with established methods like the differential staining cytotoxicity (DiSC)-assay that measures reliably the overall rate of cell death induced by anticancer drugs [6,8,11,34,37,38]. The cytotoxic drugs cytarabine, daunorubicin and mitoxantrone, commonly used in the treatment of AML were tested in both assays.

In the Casp3-test, the activation of proteases that recognize the DEVD peptide motif (DEVD-ases), especially caspase-3 was detected by measuring a fluorescence signal generated by a fluorogenic dye upon cleavage by caspase-3. It has been repeatedly demonstrated that apoptosis usually is the dominant mode of tumor cell death promoted by chemotherapy [19,39,40], and caspase-3 is a major effector protease in this process [41-44]. Our results indicate, however, that levels of caspase-3 activation, i.e. DEVD-ases, do not correlate with sensitivity or resistance to commonly employed anticancer drugs in AML blasts. Even though apoptosis is the main pathway for drug-induced cell death caspase-3 activities fail to predict the response to chemotherapy as determined by measuring ex-vivo chemosensitivity by the DiSC-assay. There was no association between LC_90_-values (DiSC-assay) and corresponding K_m_-values (Casp3-test) of cytarabine (p = 0.491), daunorubicin (p = 0.964) or mitoxantrone (p = 0.544).

The DiSC-assay measures total cell death and, therefore, integrates apoptotic and non-apoptotic death induced by chemotherapy. Moreover, the DiSC-assay was shown to identify response to therapy and also long-term survival in various hematological neoplastic diseases [8,35]. This assay has been extensively evaluated in B-CLL, AML and, also, ALL [8,11,36-38,45]. In a large series of AML patients, we have recently shown an overall predictive accuracy for the DiSC-assay of 98.2% concerning treatment response and proofed the ex-vivo chemosensitivity evaluated by the DiSC-assay as one of the strongest prognostic factors [34]. For the DiSC-assay, it has also been demonstrated that it runs more reliable than colony-forming assays that measure clonogenic growth and that results obtained by DiSC-assay are equivalent to both, the colony-forming assay and the colorimetric MTT-assay [32,36]. Based on this background, the DiSC-assay serves as an established standard method to evaluate the individual ex-vivo chemosensitivity and, also, as an appropriate reference method to evaluate and establish new ex-vivo chemosensitivity assays. Furthermore, the DiSC-assay has also been used to study various parameters influencing the apoptotic process [46,47].

Therefore, due to the lack of correlation between the Casp3-test and the DiSC-assay it must be concluded that the Casp3-test is not equivalent to the DiSC-assay and, consequently, not appropriate for the assessment of the individual ex-vivo chemosensitivity and the identification of new agents. This observation may be due to the test-system itself, but it seems more likely that it is due to the methodology of the Casp3-test to assess chemosensitivity.

Most established ex-vivo chemosensitivity assays measure total apoptotic cell death after all drugs tested have exerted their apoptotic effect. In contrast, determination of caspase-3 activation only reflects the apoptosis at a single time. However, total apoptotic cell death is the result of all apoptotic processes which occur during the whole incubation time of 96 h in the DiSC assay allowing enough time for all drugs studied to exert their apoptotic effect. It becomes clear that the intensity of apoptosis at one time point does not necessarily represent the intensity of all apoptotic processes. Moreover, anticancer drugs may also induce caspase-independent cell death and this could be neglected when only caspase-3 activities are determined [26]. It might be argued that 16 hours of incubation with cytotoxic agents in fresh leukemic cells may be too short for the evaluation of caspase-3 activity, but in all cell line experiments K_m_-values were found stabilized within 14 hours. Additionally, K_m_-values could be calculated in 92% of the Casp3-tests done on patient material, and the median K_m_-values were comparable with median LC_90_-values of the DiSC-assay implicating an adequate incubation period.

## Conclusion

In conclusion, the use of a caspase-3 activity test for the assessment of the ex-vivo chemosensitity does not appear to be suitable for the prediction of drug responses. Therefore, established tests like the DiSC-assay or the MTT-assay are still the best choice for predicting chemosensitivities of cancer cells.

## Competing interests

The company Evotec Analytical Systems, Erkrath, Germany provided funding to perform this study at our institution. The authors declare that they are not related to the company Evotec Analytical Systems in any way and that they have no financial or non-financial competing interests. Furthermore, the authors declare that the description of the results in this manuscript was not influenced by the company and that no information was withheld.

## Authors' contributions

PS conceived of the study, and participated in its design and coordination and drafted and revised extensively the manuscript. JT performed the caspase-3 activity tests and its interpretation and drafted the manuscript. TST performed the DiSC-assays and its interpretation. TS participated in the design of the study and performed the statistical analysis and helped to draft the manuscript.

## Pre-publication history

The pre-publication history for this paper can be accessed here:



## Figures and Tables

**Table 1:** Patient characteristics

Patients diagnosed with AML, no	42
Age [years]: median (range)	45 (19 – 73)
Female/male ratio, no	16/26
Successful DiSC-assays, no (%)	37/42 (88%)
Successful Casp3-tests, no (%)	42/42 (100%)
Successful single drug tests, no (%)	116/126 (92%)
DiSC-assay / Casp3-test correlation, no (%)	37/42 (88%)
Correlation between single drugs, no (%)	94/126 (75%)
FAB-groups	No
M1	3
M2	12
M3	4
M4	10
M5	5
M6	1
Secondary AML	7

**Table 2:** Median LC_90_- (DiSC-assay) and K_m_-values (Casp3-test) for cytarabine, daunorubicin and mitoxantrone (range).

Drug	LC_90 _(μg/ml)	K_m _(μg/ml)
Cytarabine	1.40 (0.06 – 147.44)	0.270 (0.062 - >20)
Daunorubicin	0.63 (0.2 – 1.59)	0.443 (0.011 - >5)
Mitoxantrone	0.235 (0.02 – 3.34)	0.222 (0.007 - >20)
